# Sex differences in disease presentation, surgical and oncological outcome of liver resection for primary and metastatic liver tumors—A retrospective multicenter study

**DOI:** 10.1371/journal.pone.0243539

**Published:** 2020-12-14

**Authors:** Eva Braunwarth, Benedikt Rumpf, Florian Primavesi, David Pereyra, Margarethe Hochleitner, Georg Göbel, Silvia Gasteiger, Philipp Gehwolf, Dietmar Öfner, Patrick Starlinger, Stefan Stättner

**Affiliations:** 1 Department of Visceral, Transplantation and Thoracic Surgery, Medical University of Innsbruck, Innsbruck, Austria; 2 Department of Surgery, Medical University of Vienna, General Hospital, Vienna, Austria; 3 Department of Internal Medicine I, Medical University of Innsbruck, Innsbruck, Austria; 4 Women´s Health Care Centre, Medical University of Innsbruck, Innsbruck, Austria; 5 Department of Medical Statistics, Informatics and Health Economics, Medical University of Innsbruck, Innsbruck, Austria; 6 Division of Hepatobiliary and Pancreatic Surgery, Department of Surgery, Mayo Clinic, Rochester, Minnesota, United States of America; 7 Department of General, Vascular and Visceral Surgery, Salzkammergut Klinikum, Vöcklabruck, Austria; University of Michigan Medical School, UNITED STATES

## Abstract

**Background:**

Sex differences are becoming of rising interest in many fields of medicine. It remains unknown whether sex has a role in postoperative and long-term outcome after hepatic resection (HR). The aim of this study was to investigate sex differences in disease presentation, surgical and oncological outcome after curative HR.

**Methods:**

Retrospective analysis of 1010 patients who underwent HR between 2005 and 2018 at two tertiary hospitals in Austria. Demographics and survival data were obtained from a prospectively maintained database. Univariate analysis was used to identify sex differences for the entire cohort and for sub-cohorts. Disease-free- and overall survival was assessed by the Kaplan-Meier estimate and results were compared by log-rank tests.

**Results:**

436 females and 574 males were analyzed. Women were younger (p<0.001), had less liver cirrhosis (p<0.001), cardiac comorbidities (p<0.001), diabetes (28 (p<0.001) and obesity (p<0.001). Type of HR and surgical management did not vary by sex. Ninety-day morbidity (p = 0.179) and -mortality (p = 0.888) were comparable. In patients with malignant disease, no differences in disease-free- and overall survival was observed, neither for the entire cohort nor for the subgroups according to tumor entity or type of resection. Only in HCC patients, females showed an inferior OS (p = 0.029).

**Conclusion:**

This study delivers new insights on the impact of sex differences in liver surgery. Despite the fact that male patients have a higher incidence of preoperative morbidities, we did not observe specific disparities in terms of immediate postoperative as well as long term oncological outcome between sexes.

## Introduction

Sex differences of diseases and outcomes have become increasingly important over the last years [1]. In this regard, sex differences were first analyzed in the field of endocrinology and reproductive medicine. Later on several clinical and experimental researchers postulated that female sex has a protective effect on infectious diseases and sepsis [2]. More recently studies also evaluated differences between women and men in the context of surgery regarding indication, therapeutic characteristics and outcome, including cardiac-, urological- and visceral procedures. Sex differences might also influence access to healthcare leading to inevitable disparities in treatment. In general emergency surgery for example, older females are more likely to get treated conservatively than older men, furthermore women are more frequently diagnosed with cancer in the emergency setting than men [3].

In colorectal cancer (CRC), disparities in disease presentation and outcomes are regularly presented, with men having higher incidence rates than females. Interestingly enough though, longitudinal cohort studies from safety net hospitals showed no differences in main oncological outcomes in CRC [4].

Sex differences which predict short-term surgical and long-term oncological outcome following hepatic resection (HR) have not been well established. The aim of this retrospective analysis from two tertiary care hospitals in Austria was to investigate the impact of sex on disease presentation, treatment, perioperative outcome and long-term survival after HR for benign and malignant (primary and metastatic) liver tumors.

## Materials and methods

### Patient selection

The clinical records of 1010 patients who underwent HR between 2001 and 2018 at the Department of Visceral, Transplantation and Thoracic Surgery at the Medical University of Innsbruck and the Department of Surgery at the Medical University of Vienna were reviewed from prospectively maintained databases. The inclusion criterion was liver resection with curative intent. The study design was approved by the Ethical Review Board (1052/2019) of the two departments. Informed consent for clinical analyses was obtained from each patient at both institutions involved and all the analyses were performed in accordance with the Declaration of Helsiniki and the local ethics policies. Diagnosis for HR included primary hepatobiliary cancer, secondary metastatic tumors and benign lesions including hepatic adenoma, focal nodular hyperplasia, hemangioma and echinococcus disease. All benign indications except echinococcus disease are consecutively summarized as “other benign lesions”. Secondary metastatic tumors were divided into colorectal cancer (CRC) and non-colorectal cancer (non-CRC), which summarizes all other secondary malignancies.

### Demographic and clinical variables

Demographic variables included age, sex, cirrhosis, diabetes, obesity (body mass index (BMI) > 30 kg/m2), cardiac or pulmonary comorbidities, chronic kidney disease and history of preoperative and postoperative chemotherapy. Clinical variables included diagnosis, extent and type of resection (according to the Brisbane 2000 Terminology of Liver Anatomy and Resections) [5], surgical approach (open or minimal invasive), preoperative portal vein embolization (PVE), intraoperative intermittent pedicle clamping (Pringle maneuver) [6], regional lymphadenectomy, bilioenteric reconstruction, concomitant vascular reconstruction, duration of surgery and intraoperative administration of red blood cell concentrates or fresh frozen plasma, estimated blood loss and duration of hospital stay.

Tumor variables such as tumor stage, resection margin status, bilobar involvement, diameter of the largest lesion, number of lesions and disease presentation metastatic malignancies (synchronous versus metachronous; cut-off: ≥6 months) were analyzed.

### Operative procedures

The hepatectomies analyzed in this study were the first resection in 945 patients, and resection for recurrence in 65 patients. In most patients who underwent segmentectomy or more extensive resection in an open approach, an ultrasonic surgical aspirator or crush-clamping technique was used for hepatic dissection. Total or selective intermittent inflow clamping and surgical drains were used according to the surgeon´s preference.

### Postoperative complications and mortality

Operative complications assessed within 90 days postoperatively included bile leakage, postoperative liver failure (POLF), acute kidney injury, hemorrhage, surgical site infection (SSI) and operative death within 90 days. Bile leakage was clinically classified according to the international study group of liver surgery (ISGLS) definition [7]. POLF was also defined according to the ISGLS definition. Acute kidney injury was defined as necessity for postoperative hemodialysis in previously non-dialytic patients. The severity of complications was assessed using the Clavien-Dindo classification graded 1 to 5 [8]. Major complications were defined as grade 3 or higher. Postoperative mortality was defined as death from any cause excluding tumor progression within 90 days after HR.

### Treatment outcome

All patients were monitored for recurrence by assessment of tumor markers and repeated chest, abdominal and pelvic computed tomography (CT) according to the centers follow up strategy (usually every 6 months for the first 5 years and then yearly thereafter), further work up with magnetic resonance imaging or Positron Emission Tomography was performed if required. Treatment outcomes were ascertained based on a prospectively maintained database and patients`clinical files. Survival data was cross-checked with the official national death registry from Statistics Austria [9].

### Statistical analysis

Nominal variables are reported as frequencies and percentages and continuous variables as medians with either interquartile range or total range, respectively. Differences in continuous clinicopathological variables between female and male patients were analyzed by the Wilcoxon signed-rank test, differences in nominal variables were investigated by chi-square or Fisher’s exact test as appropriate. The influence of sex on disease-free survival (DFS) and overall survival (OS) was estimated by the Kaplan-Meier method and the results were compared statistically by log-rank tests. Two-tailed p-values < 0.05 were considered significant and confidence intervals (CI) were reported on a 95% level. The data analysis was performed using SPSS version 24.0 (IBM, Armonk, NY, USA). The statistical methods of this study were reviewed by from Department of Medical Statistics, Informatics and Health Economics, Medical University of Innsbruck, Innsbruck, Austria.

## Results

### Patients and tumor characteristics

Demographic and clinical features of the 1010 patients stratified by sex are summarized in Table 1. There were 436 (43.2%) female and 574 (56.8%) male patients. Pathological diagnosis included hepatocellular carcinoma (HCC) in 110 cases, intrahepatic cholangiocarcinoma (ICC) in 54 cases, perihilar cholangiocarcinoma (pCC) in 106 patients, CRC in 489 patients, non-CRC in 114 cases, hydatid disease in 45 patients and other benign liver lesions in 91 cases. Benign lesions included 17 liver cysts, 19 hemangiomas, 21 adenomas, 23 focal nodular hyperplasias, 5 primary sclerosing cholangitis cases, 3 primary biliary cirrhosis cases and 3 others. Women were significantly younger with median 59 years versus 62 years in men (p<0.001). Furthermore, females were less likely to suffer from cardiac comorbidities (p<0.001), underlying liver cirrhosis (p<0.001), diabetes (p<0.001) and obesity (p<0.001).

**Table 1:** Demographic and tumor characteristics stratified by sex.

	Total (n = 1010)	Female (n = 436)	Male (n = 574)	*p*
Age (years)*	61 (19–89)	59 (29–89)	62 (19–86)	**<0.001**
Comorbidities, n (%)				
Cirrhosis, n (%)	55 (5.6)	9 (2.1)	46 (8.3)	**<0.001**
Cardiac	119 (12.1)	28 (6.6)	91 (16.4)	**<0.001**
Pulmonary	69 (7.0)	22 (5.2)	47 (8.4)	0.051
Chronic kidney disease	28 (2.8)	10 (2.3)	18 (3.2)	0.410
Diabetes	121 (12.1)	28 (6.5)	93 (16.4)	**<0.001**
Obesity (BMI>30 kg/m^2^)	165 (16.5)	47 (10.9)	118 (20.7)	**<0.001**
Neoadjuvant chemotherapy, n (%)	486 (57.9)	188 (55.6)	298 (59.5)	0.267
Adjuvant chemotherapy, n (%)	432 (57.3)	172 (58.9)	260 (56.3)	0.477
Tumor stage, n (%)				0.342
T1	97 (12.7)	32 (11.1)	65 (13.8)	
T2	164 (21.6)	56 (19.4)	108 (22.9)	
T3	413 (54.3)	164 (56.7)	249 (52.8)	
T4	87 (11.4)	37 (12.8)	50 (10.6)	
Bilobar involvement, n (%)	259 (37.3)	109 (37.8)	150 (36.9)	0.790
Synchronous disease, n (%)	370 (61.2)	153 (61.2)	217 (61.1)	0.985
Diameter of largest lesion, (mm)**	47.3 (3.0–300.0)	50.9 (3.0–300.0)	44.7 (3.0–280.0)	0.075
Number of lesions*	1 (1–15)	1 (1–15)	1 (1–12)	0.348

BMI, body mass index; mm millimeter;

* median (range),

** mean (range).

Taking other malignancies than HCC into account, men received significantly more neoadjuvant chemotherapy than women (68.9% vs. 59.2%, p = 0.007, S2 Table).

### Surgical treatment and perioperative outcome

Operative parameters are listed in Table 2. The majority of patients underwent major hepatectomy (see Table 2) with no significant differences between female (n = 215) and male (n = 286) patients (p = 0.807). Anatomical, non-anatomical and combined resections were performed at similar rates (p = 0.735). Right and extended right hepatectomy were the most common types of resection (p = 0.734). The median length of hospital stay was comparable for women and men (p = 0.399, Table 2). Significant differences were found for intermittent pedical clamping, which was more frequently used in male patients compared to females (p = 0.017) and operative time was shorter in women (258min versus 284min; p = 0.005).

**Table 2:** Surgical treatment stratified by sex.

	Total (n = 1010)	Female (n = 436)	Male (n = 574)	*p*
Major hepatectomy, n (%)	501 (49.8)	215 (49.3)	286 (50.1)	0.807
Type of resection, n (%)				0.735
anatomical	483 (48.5)	203 (47.3)	280 (49.4)	
non-anatomical	384 (38.6)	167 (38.9)	217 (38.3)	
combined procedure	129 (13.0)	59 (13.8)	70 (12.3)	
Type of hepatectomy, n (%)				0.734
Right hepatectomy	152 (24.8)	61 (23.3)	91 (26.0)	
Left hepatectomy	63 (10.3)	26 (9.9)	37 (10.6)	
Extended right hepatectomy	111 (18.1)	52 (19.8)	59 (16.9)	
Extended left hepatectomy	36 (5.9)	17 (6.5)	19 (5.4)	
Left lateral sectorectomy	69 (11.3)	34 (13.0)	35 (10.0)	
Segmentectomy	100 (16.3)	40 (15.3)	60 (17.1)	
Bisegmentectomy	77 (12.6)	30 (11.5)	47 (13.4)	
ALPPS	3 (0.5)	2 (0.8)	1 (0.3)	
Mini ALPPS	1 (0.2)	0 (0.0)	1 (0.3)	
Laparoscopic procedure, n (%)	37 (3.7)	16 (3.7)	21 (3.7)	0.980
Vascular reconstruction, n (%)	27 (2.7)	11 (2.6)	16 (2.9)	0.792
Bilioenteric reconstruction, n (%)	91 (9.0)	40 (9.2)	51 (8.9)	0.874
Pringle maneuver, n (%)	144 (14.3)	49 (11.2)	95 (16.6)	**0.017**
Portal vein embolization, n (%)	59 (5.8)	31 (7.1)	28 (4.9)	0.139
Lymphadenectomy, n (%)	136 (13.8)	58 (13.6)	78 (13.9)	0.899
Operative time (minutes)*	273 (34–1045)	258 (34–1045)	284 (35–936)	**0.005**
Red cell concentrate (300ml units)*	0 (0–28)	0 (0–28)	0 (0–26)	0.788
Fresh frozen plasma (250ml units)*	0 (0–45)	2 (0–45)	2 (0–35)	0.559
Intraoperative blood loss (ml)*	300 (45–1996)	200 (50–14450)	300 (45–21583)	0.359
Hospital stay (days)*	10 (1–119)	9 (1–119)	10 (1–118)	0.399

mm milliliter;

* median (range).

A detailed analysis of postoperative morbidity is shown in Table 3. Ninety-day morbidity (p = 0.179) and—mortality (p = 0.888) were comparable between sexes (Table 3). Most frequent causes of death were multiple-organ failure (n = 15), and POLF (n = 4). A detailed information on 90-day mortality stratified by sex is provided in the S3 Table. There was no difference in the rate of any specific complication recorded in the entire cohort. Concerning HCC patients, females were more likely to get severe complications CD ≥3 than male patients (47.6% vs. 24.7%, p = 0.038, S4 Table). This was not found for other malignancies (S5 Table). Management of postoperative complications was equally distributed, reoperation was performed in 102 patients, percutaneous drainage was performed in 113, endoscopic interventions in 45 and interventional angiography in 12 patients. Women and men achieved an equal percentage of histologically tumor free margins (p = 0.749, Table 4).

**Table 3:** Postoperative morbidity and mortality stratified by sex.

	Total (n = 1010)	Female (n = 436)	Male (n = 574)	*p*
90-day mortality, n (%)	31 (3.1)	13 (3.0)	18 (3.1)	0.888
90-d morbidity, n (%)	401 (39.9)	163 (37.6)	238 (41.8)	0.179
Severe Complications (CD ≥3), n (%)	244 (24.2)	101 (23.2)	143 (24.9)	0.520
Malignant disease only	225 (25.8)	93 (27.0)	132 (25.0)	0.492
Hemorrhage, n (%)	40 (4.0)	15 (3.5)	25 (4.4)	0.445
Bile leakage, n (%)	90 (9.0)	35 (8.1)	55 (9.7)	0.377
Postoperative liver failure, n (%)	89 (8.8)	37 (8.5)	52 (9.1)	0.731
Acute kidney injury, n (%)	28 (2.8)	9 (2.1)	19 (3.4)	0.227
Surgical site infection, n (%)	73 (7.3)	30 (6.9)	43 (7.6)	0.695
Cardiac complications, n (%)	50 (5.0)	17 (3.9)	33 (5.8)	0.174

C-D classification Clavien-Dindo classification.

**Table 4:** Disease-free and overall survival analysis stratified by sex (malignant disease only).

	Total (n = 873)	Female (n = 344)	Male (n = 529)	*p*
Tumor free margin, n (%)	762 (95.0)	310 (95.4)	452 (94.8)	0.689
Recurrence, n (%)	616 (70.6)	240 (69.8)	376 (71.1)	0.678
DFS (months)*				
Major hepatectomy (n = 442)	18 (16.2–19.7)	16 (13.1–18.9)	19 (16.9–21.0)	0.745
HCC (n = 110)	17 (12.7–21.3)	10 (0.0–23.7)	20 (16.1–23.8)	0.662
ICC (n = 54)	8 (0.0–16.5)	8 (0.0–21.7)	8 (4.1–11.9)	0.462
pCC (n = 106)	14 (12.0–16.0)	12 (6.9–17.1)	14 (8.6–19.4)	0.337
CRC (n = 489)	25 (21.1–29.0)	22 (16.0–28.1)	26 (22.2–29.8)	0.227
Non-CRC (n = 114)	23 (18.7–23.3)	30 (12.4–47.6)	18 (15.5–20.5)	0.489
OS (months)*				
Major hepatectomy (n = 442)	55 (46.2–63.8)	52 (40.9–63.1)	59 (49.8–68.2)	0.636
HCC (n = 110)	63 (50.6–75.4)	35 (26.9–43.1)	66 (58.0–74.0)	**0.029**
ICC (n = 54)	37 (25.9–48.0)	38 (14.8–61.2)	36 (30.6–41.4)	0.532
pCC (n = 106)	25 (18.9–31.1)	23 (15.0–31.1)	25 (116.2–33.8)	0.949
CRC (n = 489)	75 (66.7–83.3)	69 (57.3–80.7)	78 (66.9–89.1)	0.176
Non-CRC (n = 114)	102 (72.7–131.3)	113 (92.0–134.0)	59 (35.0–83.0)	0.170

DFS, disease-free survival; HCC, hepatocellular carcinoma; ICC, intrahepatic cholangiocellular carcinoma; pCC, perihilar cholangiocellular carcinoma; CRC, colorectal cancer; OS, overall survival;

* Median (95% confidence interval).

### Survival analysis

Of the 873 patients with malignant diagnosis, 616 patients (70.6%) developed tumor recurrence at a median follow up of 28 months (range 3–103).

There was no difference in median DFS between female and male patients (20 vs. 22 months, p = 0.586). In addition, we found comparable results regarding tumor entities and type of resection (Table 4, S1 Fig).

To analyze the effect of sexual hormones on oncological outcome, the cohort was stratified into age ≤ 55 and > 55 years groups. For the three most frequent cancers (HCC, pCC, CRC), no difference in median DFS in the analyzed tumor types was found (S6 Table).

Comparing the tumor stages for the various cancers, female patients with HCC in T2 stage had an inferior DFS (p<0.001), whereas no differences were observed in the pCC and CRC population (S6 Table).

**Fig 1:**
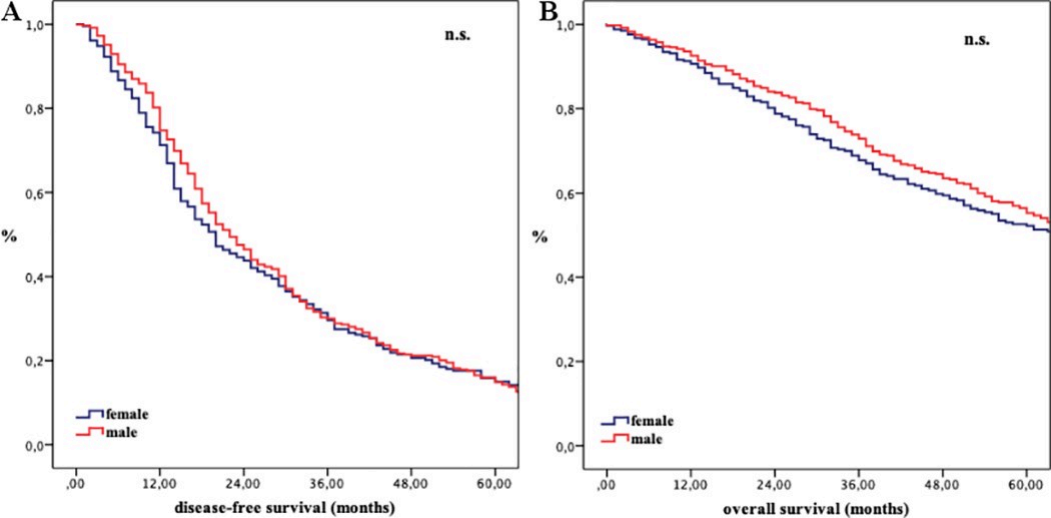
A) Disease-free survival of malignant tumors stratified by sex; B) Overall survival of malignant tumors stratified by sex; n.s. not significant.

Considering the entire cohort with malignant disease, the 1-, 3- and 5-year DFS rates were 74.2%, 31.3% and 15.9% for women and 80.4%, 30.3% and 16.0% for men, respectively (Fig 1A).

The median OS for malignant diagnosis was 65 months for female and 67 months for male patients (p = 0.331, Fig 1B). Subgroup analysis stratified by tumor entities and major hepatectomy revealed no significant results. Only for HCC patients, females showed an inferior OS (p = 0.029, Table 4, S2 Fig). In the age ≤ 55 cohort stratified by tumor entity female patients suffering from HCC had a worse DFS (p = 0.014). This effect vanished in the age > 55 cohort.

In line with DFS, female HCC patients in T2 stage had an inferior OS compared to males (p = 0.008). Subgroup analysis for OS in pCC and CRC patients showed comparable results. Detailed data is shown in S7 Table.

## Discussion

This retrospective study evaluated sex differences in disease presentation, treatment, short-term surgical outcome and long-term oncological outcome of a consecutive cohort of patients undergoing HR for benign and malignant liver tumors at the two largest university medical centers in Austria. We purposely did not define or exclude sub-cohorts of patients, in order to analyze a cohort representing realistic overall surgical care. In most aspects, no differences in treatment and perioperative and oncological outcomes after curative liver resection between female and male patients could be observed. While this itself is an important message, several significant differences related to sex emerged.

Men were diagnosed at a significantly older age than women, had significantly more cardiac comorbidities, underlying liver cirrhosis, diabetes and obesity. Interestingly this higher risk features did not translate into higher perioperative morbidity. Especially, men who suffer from more cardiac comorbidities, did not show a higher rate of cardiac complications. In contrast, a Norwegian national cohort study found that male sex is a potential risk factor for revisional surgery after liver resection [10]. HCC patients are thought to be associated with impaired liver functional reserve. Interestingly, no differences were found in developing POLF in HCC patients. Both aspects might in fact be explainable by the high level of perioperative care and facilitation of enhanced recovery programs in both our units, diminishing the classical non-surgical complication rates [11, 12].

General aspects of management, including type and extent of HR, lymph node dissection, PVE, intraoperative transfusion and blood loss did not vary by sex. This is probably not surprising as the choice of treatment and extent of surgery is generally not triggered by sex. Except the mentioned parameters, female patients had a shorter operative time and pringle maneuver was used less frequent. Possible reasons for this could be the slightly higher rate of benign tumors like adenomas and focal nodular hyperplasia among women, that are typically related to female sex [13] and the higher incidence of cirrhosis in male patients.

In terms of oncological outcome, we found similar disease presentations and tumor distributions with no differences in DFS and OS. Several studies have investigated sex differences in disease characteristics, mostly in HCC and CRC patients. Ladenheim et al. found that although women were older at time of diagnosis, they less likely had advanced liver disease or tumor burden [14]. While in our cohort men suffered more frequently from diabetes, Yokoyama et al. could not find differences between males and females in the proportion of Asian patients with diabetes [15]. Additionally Farinati et al. demonstrated that HCC in females is more frequently unifocal and of smaller size and has a lower prevalence of portal thrombosis and extrahepatic metastases [16]. A larger series from Zhang et al. found no difference in early recurrence rate after HR between sexes, whereas men had significantly greater late recurrences (>2 years) and rates of cancer-specific mortality after resection for HCC [17]. In our European cohort survival of female patients suffering HCC was significantly worse, keeping in mind quite low numbers of cases and the apparent differences in underlying liver disease (increased incidence of cirrhosis in men). Accordingly, this explorative finding will need further assessment in larger patient cohorts. Besides HCC patients, sex differences are also described for CRC patients. Quirt and colleagues evaluated sex differences in 7249 patients who underwent resection for CRC. In their analysis, the authors could not observe differences in stage of cancer between men and women [18]. Other authors described women being older at resection, having less likely metastatic disease and earlier T-categories, which is contrary to other reports which describe women to have tumors with more advanced stage, higher-grade disease and more aggressive biological characteristics [19–22]. The reason for women being diagnosed at younger age and earlier tumor stage may result from men being less frequently diagnosed by routine screening [14]. Especially for early detection of CRC, screening programs intend to detect lesions at a premalignant stage [23]. It is known that perception and psychological barriers towards CRC screening differ between women and men. Women tend to procrastinate CRC screening due to associated distress, while men tend to displace negative views while strongly deferring the task of complete screening [24].

In line with our findings, several authors described primary malignant liver tumors to be more common in men [25, 26].

Surgical management did not differ between sexes in our cohort, women achieved a comparable rate of histological tumor free margins. Overall 90-day morbidity and -mortality did not differ by sex as well, this is not in line with other series. Offner et al. identified male gender as an independent risk factor for the development of severe infections in surgical patients [27]. Some researchers found male gender as a major negative risk factor influencing the clinical course of patients being affected by trauma or sepsis [28, 29]. One potential explanation is that female patients have a more effective immune response following extensive abdominal operations [2, 30]. It has been previously described that sex hormones modulate the immune and cardiovascular responses following trauma [2].

Bachellier et al. found male sex as an independent risk factor for POLF in patients who underwent major hepatectomy with combined portal vein resection [31] probably due to a different pathophysiologic response to hepatic stress in men. Yokoyama et al. indicated that female hormones promote hepatocyte proliferation, whereas male hormones suppress this crucial mechanism [15]. Anyway, we could not confirm these results in our large cohort which showed no differences in POLF besides equal distribution of major resections.

Regarding DFS between men and women, it is reported that immunological differences between sexes can influence oncological outcome. Previous reports indicated that tumor progression could be affected by the inflammatory response and the immune system [32]. An increased systematic inflammatory response is known to be associated with an inferior survival independent of the stage of the tumor [33, 34]. Campbell-Thompson et al. proposed that female sex hormones have a protective immunologic effect on the inflammatory response in CRC patients [35]. In HCC, an androgen-sensitive tumor, sex hormones can promote tumor proliferation [36, 37]. If estrogen levels have a benefit on survival, one would expect that survival differences between women and men would be limited to younger patients. Nevertheless, this theory could not be observed in our female cohort of <55 years patients.

Limitations of this study besides its retrospective nature, include the rather small size of the study population in regard to the heterogeneity of tumor entities. Furthermore, the two centers participating in this study represent the two largest hepatobiliary centers in Austria, hence there might be a selection bias to more complex patients. For example, primary care hospitals do not perform resections of Klatskin tumors. Besides this, patients with smaller tumors are often treated with stereotactic radiofrequency ablation and not with surgery [38]. This is also reflected in the high rate of major resections (49.8%). Nevertheless, 1010 patients are a convincing number to make conclusive analyses for postoperative morbidity after HR between the two sexes.

## Conclusion

This study delivers data on sex differences in patients after HR, a topic that has not been studied extensively. Besides significant differences in patient risk factors, no specific disparity in outcome between male and female patients was detected. Gender does not typically trigger therapeutic decisions, although it does influence the incidence and disease presentation of many cancers. In times of individualized medicine and treatment concepts, future prospective and large-scale studies should focus on sex differences to obtain strong gender specific data on surgical and oncological outcome and to evaluate the role of sex hormones in different cancer types.

## Supporting information

S1 Table Demographic and tumor characteristics in HCC patients stratified by sex. BMI, body mass index; mm millimeter; * median (range), ** mean (range).
(DOCX)

S2 Table Demographic and tumor characteristics of patients with malignancy except HCC. BMI, body mass index; mm millimeter; * median (range), ** mean (range).
(DOCX)

S3 Table Cause of death within 90 days stratified by sex. (DOCX)

S4 Table Postoperative morbidity and mortality in HCC patients stratified by sex. C-D Clavien-Dindo classification.
(DOCX)

S5 Table Postoperative morbidity and mortality of patients with malignancy except HCC. C-D Clavien-Dindo classification.
(DOCX)

S6 Table Sub-group analysis: Disease-free survival stratified by sex, age, tumor entity and tumor stage. DFS, disease-free survival; HCC, hepatocellular carcinoma; carcinoma; pCC, perihilar cholangiocellular carcinoma; CRC, colorectal cancer; * Median (95% confidence interval), n.c., not calculable.
(DOCX)

S7 Table Sub-group analysis: Overall survival stratified by sex, age, tumor entity and tumor stage. OS, overall survival; HCC, hepatocellular carcinoma; carcinoma; pCC, perihilar cholangiocellular carcinoma; CRC, colorectal cancer; * Median (95% confidence interval), n.c., not calculable.
(DOCX)

S1 Fig Disease-free survival stratified by tumor entities and sex. A) female patients, B) male patients, HCC hepatocellular carcinoma, ICC intrahepatic cholangiocarcinoma, pCC perihilar cholangiocarcinoma, CRC colorectal cancer, Non CRC non colorectal secondary liver tumors.
(TIF)

S2 Fig Overall survival stratified by tumor entities and sex. A) female patients, B) male patients, HCC hepatocellular carcinoma, ICC intrahepatic cholangiocarcinoma, pCC perihilar cholangiocarcinoma, CRC colorectal cancer, non CRC non colorectal secondary liver tumors.
(TIF)

S1 Dataset Minimal anonymized dataset. (SAV)
