# Supplementary material for: Sex differences in disease presentation, surgical and oncological outcome of liver resection for primary and metastatic liver tumors—A retrospective multicenter study
Source: PLoS One. 2020 Dec 14;15(12):e0243539. doi: 10.1371/journal.pone.0243539 (PMC7735568; doi:10.1371/journal.pone.0243539)
Supplement: S1 Table — BMI, body mass index; mm millimeter; * median (range), ** mean (range). (DOCX) [file pone.0243539.s001.docx]

|  | Total  (n=110) | Female  (n=21) | Male  (n=89) | *p* |  |  |
| --- | --- | --- | --- | --- | --- | --- |
| Age (years)^*^ | 66 (32 - 86) | 63 (37–78) | 67 (32–86) | 0.094 |  |  |
| Comorbidities, n (%) |  |  |  |  |  |  |
| Cirrhosis, n (%) | | 44 (41.1) | 6 (28.6) | 38 (44.2) | 0.192 | |
| Cardiac | | 35 (34.0) | 7 (35.0) | 28 (33.7) | 0.915 | |
| Pulmonary | | 14 (13.2) | 1 (5.0) | 13 (15.1) | 0.229 | |
| Chronic kidney disease | | 8 (7.5) | 1 (4.8) | 7 (8.1) | 0.598 | |
| Diabetes | | 29 (26.6) | 6 (28.6) | 23 (26.1) | 0.821 | |
| Obesity (BMI>30 kg/m^2^) | | 28 (26.4) | 4 (19.0) | 24 (28.2) | 0.392 | |
| Neoadjuvant chemotherapy, n (%) | 17 (16.8) | 6 (31.6) | 11 (13.4) | 0.057 |  |  |
| Adjuvant chemotherapy, n (%) | 18 (19.8) | 6 (35.3) | 12 (16.2) | 0.075 |  |  |
| Tumor stage, n (%) |  |  |  | **0.011** |  |  |
| T1 | 27 (28.1) | 1 (5.9) | 26 (32.9) |  |  |  |
| T2 | 38 (39.6) | 6 (35.3) | 32 (40.5) |  |  |  |
| T3 | 23 (24.0) | 9 (52.9) | 14 (17.7) |  |  |  |
| T4 | 8 (8.3) | 1 (5.9) | 7 (8.9) |  |  |  |
| Bilobar involvement, n (%) | 23 (21.7) | 6 (28.6) | 17 (20.0) | 0.393 |  |  |
| Diameter of largest lesion,  (mm)^**^ | 70 (9–270) | 90 (11–270) | 65 (9–200) | 0.055 |  |  |
| Number of lesions^*^ | 1 (1–4) | 1 (1–3) | 1 (1–4) | 0.198 |  |  |
