# Supplementary material for: Sex differences in disease presentation, surgical and oncological outcome of liver resection for primary and metastatic liver tumors—A retrospective multicenter study
Source: PLoS One. 2020 Dec 14;15(12):e0243539. doi: 10.1371/journal.pone.0243539 (PMC7735568; doi:10.1371/journal.pone.0243539)
Supplement: S2 Table — BMI, body mass index; mm millimeter; * median (range), ** mean (range). (DOCX) [file pone.0243539.s002.docx]

|  | Total  (n=763) | Female  (n=323) | Male  (n=440) | *p* |  |  |
| --- | --- | --- | --- | --- | --- | --- |
| Age (years)^*^ | 62 (19 - 89) | 61 (19–89) | 62 (23–86) | 0.052 |  |  |
| Comorbidities, n (%) |  |  |  |  |  |  |
| Cirrhosis, n (%) | | 11 (1.5) | 3 (0.9) | 8 (1.9) | 0.299 | |
| Cardiac | | 74 (9.9) | 16 (5.0) | 58 (13.6) | **<0.001** | |
| Pulmonary | | 48 (6.4) | 17 (5.4) | 31 (7.2) | 0.320 | |
| Chronic kidney disease | | 14 (1.9) | 5 (1.6) | 9 (2.1) | 0.604 | |
| Diabetes | | 85 (11.2) | 20 (6.2) | 65 (14.9) | **<0.001** | |
| Obesity (BMI>30 kg/m^2^) | | 126 (16.6) | 34 (10.6) | 92 (20.9) | **<0.001** | |
| Neoadjuvant chemotherapy, n (%) | 476 (64.8) | 181 (59.2) | 286 (68.9) | **0.007** |  |  |
| Adjuvant chemotherapy, n (%) | 414 (62.4) | 166 (60.4) | 248 (63.9) | 0.352 |  |  |
| Tumor stage, n (%) |  |  |  | 0.775 |  |  |
| T1 | 69 (10.4) | 30 (11.0) | 39 (9.9) |  |  |  |
| T2 | 126 (19.0) | 50 (18.4) | 76 (19.4) |  |  |  |
| T3 | 390 (58.7) | 156 (57.4) | 234 (59.7) |  |  |  |
| T4 | 79 (11.9) | 36 (13.2) | 43 (11.0) |  |  |  |
| Bilobar involvement, n (%) | 120 (44.3) | 47 (37.9) | 73 (49.7) | 0.052 |  |  |
| Diameter of largest lesion,  (mm)^**^ | 40 (3–300) | 42 (3–300) | 39 (2–280) | 0.477 |  |  |
| Number of lesions^*^ | 2 (1–15) | 2 (1–15) | 2 (1–12) | 0.560 |  |  |
