# Supplementary material for: Sex differences in disease presentation, surgical and oncological outcome of liver resection for primary and metastatic liver tumors—A retrospective multicenter study
Source: PLoS One. 2020 Dec 14;15(12):e0243539. doi: 10.1371/journal.pone.0243539 (PMC7735568; doi:10.1371/journal.pone.0243539)
Supplement: S3 Table — (DOCX) [file pone.0243539.s003.docx]

|  | Total  (n=31) | Female  (n=13) | Male  (n=18) |
| --- | --- | --- | --- |
| Multiple organ failure, n (%) | 15 (48.4) | 6 (19.4) | 9 (29.0) |
| Tumor progression, n (%) | 3 (9.7) | 2 (6.5) | 1 (3.2) |
| Hemorrhage, n (%) | 1 (3.2) | 0 (0.0) | 1 (3.2) |
| Pneumonia, n (%) | 3 (9.7) | 2 (6.5) | 1 (3.2) |
| Subarachnoidal bleeding, n (%) | 2 (6.5) | 1 (3.2) | 1 (3.2) |
| Postoperative liver failure, n (%) | 4 (12.9) | 1 (3.2) | 3 (9.7) |
| Myocardial infarction, n (%) | 3 (9.7) | 1 (3.2) | 2 (6.5) |
