# Supplementary material for: Sex differences in disease presentation, surgical and oncological outcome of liver resection for primary and metastatic liver tumors—A retrospective multicenter study
Source: PLoS One. 2020 Dec 14;15(12):e0243539. doi: 10.1371/journal.pone.0243539 (PMC7735568; doi:10.1371/journal.pone.0243539)
Supplement: S4 Table — C-D Clavien-Dindo classification. (DOCX) [file pone.0243539.s004.docx]

|  | Total  (n=110) | Female  (n=21) | Male  (n=89) | *p* |
| --- | --- | --- | --- | --- |
| 90-day mortality, n (%) | 5 (4.5) | 0 (0.0) | 5 (5.6) | 0.266 |
| 90-d morbidity, n (%) | 47 (43.5) | 12 (57.1) | 35 (40.2) | 0.161 |
| Severe Complication (CD ≥ 3), n (%) | 32 (29.1) | 10 (47.6) | 22 (24.7) | **0.038** |
| Hemorrhage, n (%) | 10 (9.5) | 4 (19.0) | 6 (7.1) | 0.096 |
| Bile leakage, n (%) | 9 (8.4) | 3 (14.3) | 6 (7.0) | 0.279 |
| Postoperative liver failure, n (%) | 15 (13.6) | 5 (23.8) | 10 (11.2) | 0.131 |
| Acute kidney injury, n (%) | 5 (4.7) | 0 (0.0) | 5 (5.9) | 0.255 |
| Surgical site infection, n (%) | 11 (10.4) | 2 (9.5) | 9 (10.6) | 0.886 |
| Cardiac complication, n (%) | 4 (3.8) | 0 (0.0) | 4 (4.7) | 0.311 |
