# Supplementary material for: Sex differences in disease presentation, surgical and oncological outcome of liver resection for primary and metastatic liver tumors—A retrospective multicenter study
Source: PLoS One. 2020 Dec 14;15(12):e0243539. doi: 10.1371/journal.pone.0243539 (PMC7735568; doi:10.1371/journal.pone.0243539)
Supplement: S5 Table — C-D Clavien-Dindo classification. (DOCX) [file pone.0243539.s005.docx]

|  | Total  (n=763) | Female  (n=323) | Male  (n=440) | *p* |
| --- | --- | --- | --- | --- |
| 90-day mortality, n (%) | 24 (3.1) | 13 (4.0) | 11 (2.5) | 0.233 |
| 90-d morbidity, n (%) | 318 (41.9) | 130 (40.5) | 188 (42.9) | 0.504 |
| Severe Complication (CD ≥ 3), n (%) | 193 (25.3) | 83 (25.7) | 110 (25.0) | 0.827 |
| Hemorrhage, n (%) | 25 (3.3) | 10 (3.1) | 15 (3.4) | 0.818 |
| Bile leakage, n (%) | 73 (9.7) | 27 (8.5) | 46 (10.6) | 0.338 |
| Postoperative liver failure, n (%) | 67 (8.8) | 30 (9.3) | 37 (8.5) | 0.693 |
| Acute kidney injury, n (%) | 20 (2.6) | 8 (2.5) | 12 (2.8) | 0.836 |
| Surgical site infection, n (%) | 56 (7.4) | 24 (7.5) | 32 (7.3) | 0.924 |
| Cardiac complication, n (%) | 39 (5.2) | 13 (4.1) | 26 (6.0) | 0.247 |
