# Supplementary material for: Sex differences in disease presentation, surgical and oncological outcome of liver resection for primary and metastatic liver tumors—A retrospective multicenter study
Source: PLoS One. 2020 Dec 14;15(12):e0243539. doi: 10.1371/journal.pone.0243539 (PMC7735568; doi:10.1371/journal.pone.0243539)
Supplement: S6 Table — DFS, disease-free survival; HCC, hepatocellular carcinoma; carcinoma; pCC, perihilar cholangiocellular carcinoma; CRC, colorectal cancer; * Median (95% confidence interval), n.c., not calculable. (DOCX) [file pone.0243539.s006.docx]

|  | Total | Female | Male | *p* |
| --- | --- | --- | --- | --- |
| DFS (months)* for sub-group age > 55 years | | | | |
| HCC (n=63) | 20 (14.9–25.1) | 20 (0.0–40.5) | 20 (14.8–25.2) | 0.600 |
| pCC (n=39) | 14 (11.8–16.2) | 10 (6.1–13.9) | 14 (10.9–17.1) | 0.726 |
| CRC (n=271) | 29 (25.5–32.5) | 27 (17.4–36.6) | 30 (25.4–34.5) | 0.229 |
| DFS (months)* for sub-group age ≤ 55 years | | | | |
| HCC (n=17) | 13 (2.9–23.1) | 7 (2.7–11.2) | 17 (5.1–28.8) | 0.072 |
| pCC (n=18) | 15 (10.9–19.1) | 13 (3.3–22.7) | 18 (14.5–21.5) | 0.153 |
| CRC (n=96) | 20 (17.8–22.2) | 20 (18.9–21.1) | 19 (15.0–23.0) | 0.917 |
|  |  |  |  |  |
| DFS (months)* for sub-group HCC | | | | |
| Tumor stage |  |  |  |  |
| T1 (n=17) | 19 (15.5–42.5) | 34 (34.0–34.0) | 24 (12.8–35.2) | 0.922 |
| T2 (n=31) | 20 (10.2–29.8) | 5 (4.3–7.0) | 22 (15.1–28.9) | **<0.001** |
| T3 (n=22) | 7 (2.4–11.6) | 10 (1,2–18.8) | 6 (2.5–9.5) | 0.197 |
| T4 (n=3) | 6 (1.2–10.8) | n.c. | 6 (1.2–10.8) | n.c. |
|  |  |  |  |  |
| DFS (months)* for sub-group pCC | | | | |
| Tumor stage |  |  |  |  |
| T1 (n=8) | 15 (6.7–23.3) | 48 (48.0–48.0) | 15 (12.4–17.5) | 0.155 |
| T2 (n=24) | 11 (6.2–15.8) | 6 (0.3–11.7) | 12 (0.1–23.9) | 0.058 |
| T3 (n=16) | 11 (8.4–13.6) | 10 (6.9–13.1) | 11 (0.0–24.2) | 0.185 |
| T4 (n=4) | 14 (10.1–17.9) | 15 (0.9–15.0) | 14 (9.2–17.9) | 0.918 |
|  |  |  |  |  |
| DFS (months)* for sub-group CRC | | | | |
| Tumor stage |  |  |  |  |
| T1 (n=8) | 35 (0.0–72.4) | 35 (19.1–83.8) | 30 (0.00–63.6) | 0.896 |
| T2 (n=38) | 27 (21.0–33.0) | 37 (15.0–59.0) | 24 (18.0–30.0) | 0.487 |
| T3 (n=246) | 27 (22.2–31.7) | 23 (14.0–32.0) | 29 (23.8–34.1) | 0.504 |
| T4 (n=51) | 19 (16.9–22.0) | 14 (12.4–15.6) | 20 (17.5–22.5) | 0.124 |
