# Supplementary material for: Sex differences in disease presentation, surgical and oncological outcome of liver resection for primary and metastatic liver tumors—A retrospective multicenter study
Source: PLoS One. 2020 Dec 14;15(12):e0243539. doi: 10.1371/journal.pone.0243539 (PMC7735568; doi:10.1371/journal.pone.0243539)
Supplement: S7 Table — OS, overall survival; HCC, hepatocellular carcinoma; carcinoma; pCC, perihilar cholangiocellular carcinoma; CRC, colorectal cancer; * Median (95% confidence interval), n.c., not calculable. (DOCX) [file pone.0243539.s007.docx]

|  | Total | Female | Male | *p* |
| --- | --- | --- | --- | --- |
| OS (months)* for sub-group age > 55 years | | | | |
| HCC (n=89) | 63 (51.4–74.5) | 39 (28.3–49.7) | 63 (54.9–71.1) | 0.192 |
| pCC (n=78) | 25 (18.1–32.0) | 23 (15.0–31.0) | 25 (14.9–35.1) | 0.543 |
| CRC (n=354) | 76 (66.1–85.9) | 76 (56.8–95.2) | 75 (63.3–86.7) | 0.583 |
| OS (months)* for sub-group age ≤ 55 years | | | | |
| HCC (n=20) | 101 (14.2–187.9) | 35 (10.2–59.8) | 101 (15.1–186.9) | **0.014** |
| pCC (n=18) | 27 (11.9–42.1) | 16 (1.2–30.8) | 29 (0.00–70.6) | 0.158 |
| CRC (n=96) | 68 (50.1–85.9) | 56 (41.7–70.3) | 83 (41.7–124.8) | 0.094 |
|  |  |  |  |  |
| OS (months)* for sub-group HCC | | | | |
| Tumor stage |  |  |  |  |
| T1 (n=27) | 62 (45.3–78.7) | 39 (0.0–39.0) | 62 (51.4–72.6) | 0.315 |
| T2 (n=38) | 68 (42.9–93.1) | 19 (0.0–48.4) | 86 (53.9–11.1) | **0.008** |
| T3 (n=23) | 34 (27.3–40.7) | 30 (0.0–64.7) | 42 (24.6–59.5) | 0.424 |
| T4 (n=8) | 35 (0.0–79.7) | 35 (35.0–35.0) | 101 (32.8–113.2) | 0.738 |
| OS (months)* for sub-group pCC | | | | |
| Tumor stage |  |  |  |  |
| T1 (n=17) | 98 (0.0–202.2) | 98 (98.0–98.0) | 27 (15.5–94.5) | 0.585 |
| T2 (n=42) | 27 (13.2–40.8) | 30 (1.4–58.6) | 21 (5.0–37.0) | 0.603 |
| T3 (n=27) | 14 (11.5–16.5) | 14 (12.1–15.9) | 19 (0.0–41.7) | 0.569 |
| T4 (n=5) | 25 (6.7–43.3) | 3 (n.c) | 25 (n.c.) | 0.221 |
| OS (months)* for sub-group CRC | | | | |
| Tumor stage |  |  |  |  |
| T1 (n=11) | 81 (22.0–139.9) | 56 (21.5–90.4) | 81 (9.2–152.8) | 0.397 |
| T2 (n=51) | 121 (98.8–143.2) | 124 (53.7–194.2) | 106 (83.8–128.2) | 0.429 |
| T3 (n=331) | 73 (63.0–83.0) | 68 (54.2–81.8) | 76 (61.8–90.2) | 0.261 |
| T4 (n=60) | 53 (36.6–69.4) | 41 (23.7–58.3) | 58 (47.4–68.6) | 0.070 |
