# Supplementary figures and images for: Sex differences in disease presentation, surgical and oncological outcome of liver resection for primary and metastatic liver tumors—A retrospective multicenter study
Source: PLoS One. 2020 Dec 14;15(12):e0243539. doi: 10.1371/journal.pone.0243539 (PMC7735568; doi:10.1371/journal.pone.0243539)

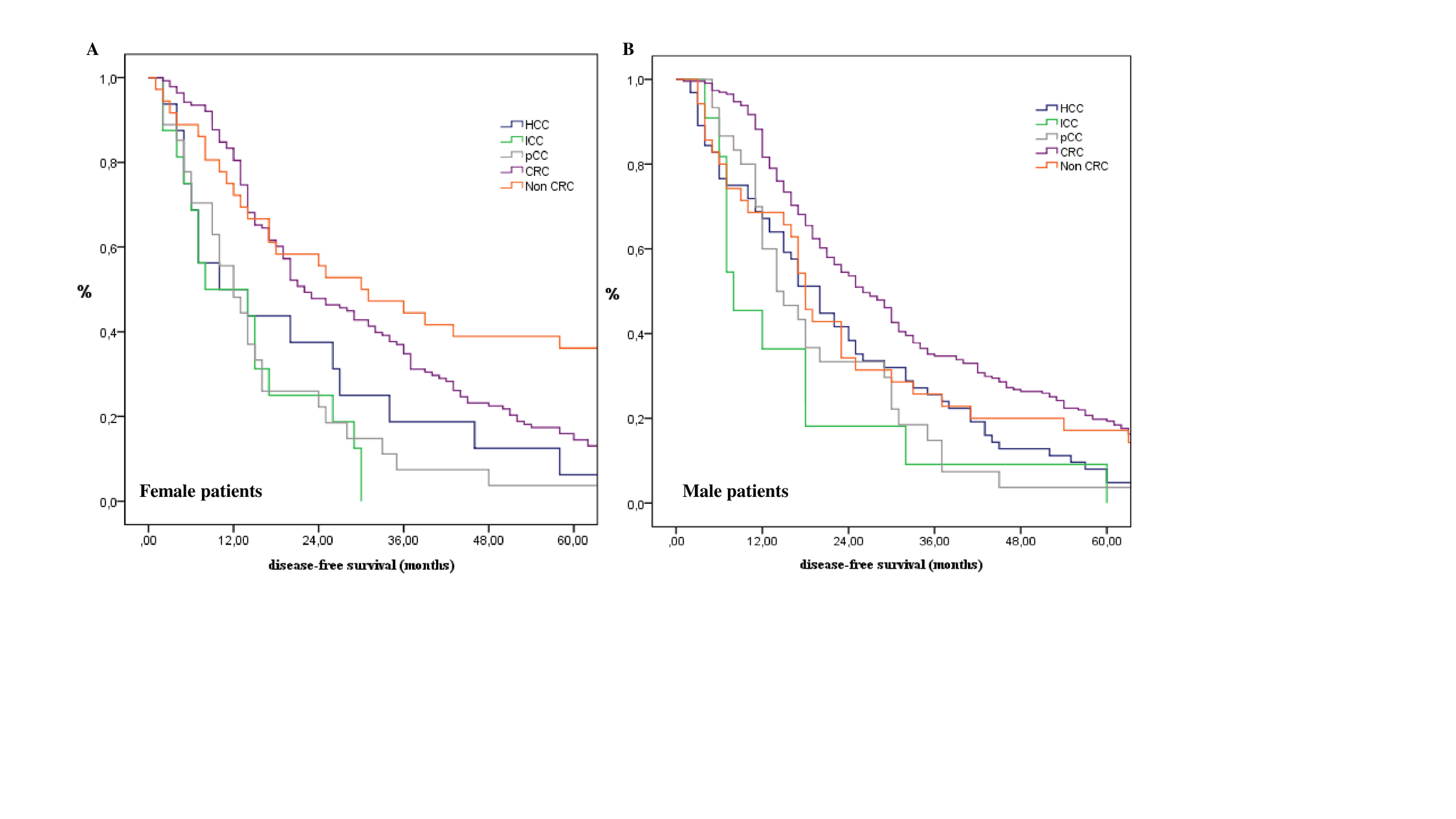

Supplement: S1 Fig — A) female patients, B) male patients, HCC hepatocellular carcinoma, ICC intrahepatic cholangiocarcinoma, pCC perihilar cholangiocarcinoma, CRC colorectal cancer, Non CRC non colorectal secondary liver tumors. (TIF) [file pone.0243539.s008.tif]

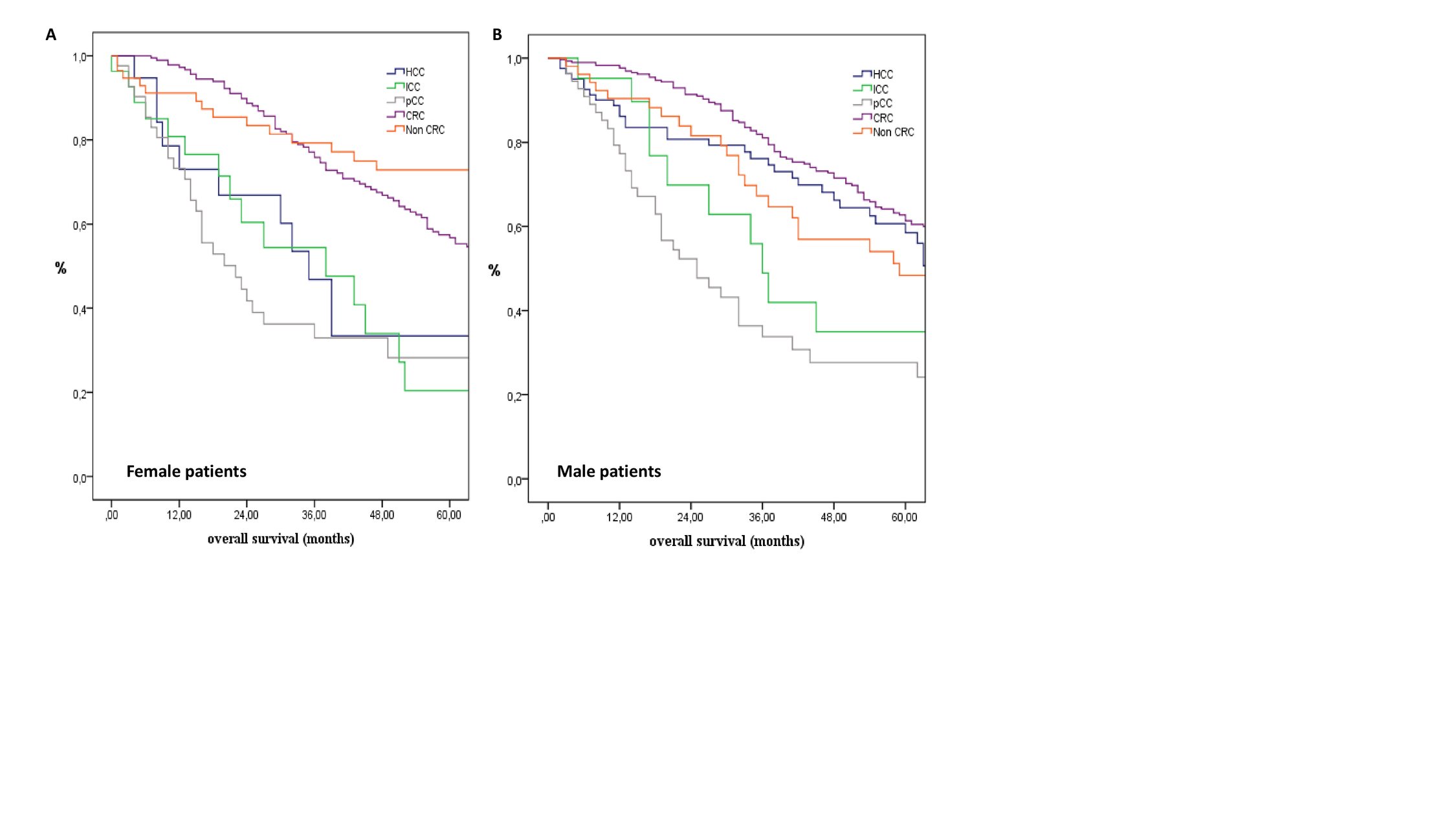

Supplement: S2 Fig — A) female patients, B) male patients, HCC hepatocellular carcinoma, ICC intrahepatic cholangiocarcinoma, pCC perihilar cholangiocarcinoma, CRC colorectal cancer, non CRC non colorectal secondary liver tumors. (TIF) [file pone.0243539.s009.tif]
